# Pilot proteomic analysis of immune dysregulation in dengue with prior SARS-CoV-2 infection

**DOI:** 10.3389/fimmu.2025.1696179

**Published:** 2026-01-08

**Authors:** Elizabeth Cruz-Altamirano, Miguel Angel Mayoral-Chávez, Luis Román Ramírez-Palacios, Luz Maria Quirino-Vela, Diego Sait Cruz-Hernández, Sergio Roberto Aguilar-Ruíz, Juan Alpuche

**Affiliations:** 1 Facultad de Medicina y Cirugía, Universidad Autonoma “Benito Juárez” de Oaxaca, Oaxaca, Mexico; 2 Laboratorio de Virología y Biología Molecular, Laboratorio Estatal de Salud Pública de Oaxaca, Oaxaca, Mexico; 3 Laboratorio de Biodiversidad, Escuela de Ciencias, Universidad Autónoma “Benito Juárez” de Oaxaca (UABJO), Oaxaca, Mexico

**Keywords:** cross-reactivity, dengue, immune dysregulation, protein expression, SARS-CoV-2

## Abstract

**Background:**

In regions where dengue and COVID-19 co-circulate, cross-reactive antibodies elicited by SARS-CoV-2 may exacerbate dengue severity. This exploratory, cross-sectional, observational pilot study evaluated whether prior SARS-CoV-2 exposure modulated dengue pathogenesis through proteomic profiling and *in vitro* assays, identifying putative biomarkers and immune pathways for further validation.

**Methods:**

Eighteen participants from Oaxaca, Mexico, were prospectively stratified into four cohorts: healthy controls (CG), dengue with anti-SARS-CoV-2 IgG (NS1/SARS_IgG), dengue with both anti-SARS-CoV-2 and anti-dengue IgG (NS1/SARS-DENV_IgG), and dengue with anti-dengue IgG only (NS1/DENV_IgG). The serum levels of IL-6 and C-reactive protein (CRP) were quantified, and proteomic analysis was performed using label-free Liquid Chromatography-Tandem Mass Spectrometry (LC–MS/MS). *In vitro* antibody-dependent enhancement (ADE) assays were performed using the DENV-1 and K562 cells.

**Results:**

The NS1/DENV_IgG group exhibited the most severe clinical presentation, including 39 °C fever and 50% thrombocytopenia. In contrast, the NS1/SARS-DENV_IgG group presented milder symptoms. IL-6 and CRP concentrations peaked in the NS1/SARS_IgG group (18.3 ± 15.6 pg/mL and 51.6 ± 29.6 mg/L, respectively). Proteomic profiling identified 279 high-confidence proteins and 18 differentially expressed proteins (DEPs). The NS1/SARS-DENV_IgG group showed enrichment in the complement/coagulation pathways (p = 0.016) and TGF-β signaling (p = 0.022). DEPs, including thrombospondin-1 and PRG2, have been implicated in Th2 polarization and ADE-like immune dysregulation. ADE assays confirmed that anti-SARS-CoV-2 serum enhanced DENV replication *in vitro*.

**Conclusions:**

Prior SARS-CoV-2 exposure may potentiate dengue severity via cross-reactive antibody-mediated immune modulation, skewing toward Th2 responses and enhancing viral replication. These findings suggest novel candidate biomarkers for stratifying the dengue risk in co-endemic regions. However, the study’s exploratory nature, small cohort (n = 18), pooled proteomic methodology, and limited clinical characterization necessitate validation in larger longitudinal studies.

## Introduction

Dengue virus (DENV), a flavivirus endemic to tropical and subtropical regions, causes an estimated 284–528 million infections annually (1). Clinical manifestations range from asymptomatic or mild febrile illness to severe dengue, characterized by vascular leakage, hemorrhagic phenomena, and multi-organ dysfunction (2). The World Health Organization (WHO) classifies dengue into three categories based on severity: dengue without warning signs, dengue with warning signs, and severe dengue (3). Risk factors for dengue severity include advanced age, underlying comorbidities (e.g., diabetes mellitus and cardiovascular disease), secondary infections involving heterologous DENV serotypes (4), and immune-mediated mechanisms such as antibody-dependent enhancement (ADE). In ADE, pre-existing non-neutralizing antibodies from prior flavivirus exposure facilitate viral entry into Fc receptor-bearing cells (5–8). This process increases viral replication, triggers cytokine storms, and exacerbates vascular pathology.

Dengue infection typically progresses through three clinical phases: acute (febrile), critical, and recovery. The acute phase (days 1–7 post-infection) is marked by abrupt-onset high fever, myalgia, arthralgia, retro-orbital pain, headache, and mild hemorrhagic signs. The critical phase begins around defervescence (temperature decline to ≤37.5 °C) and is associated with increased vascular permeability resulting in plasma leakage, hemoconcentration, thrombocytopenia, leukopenia, elevated hepatic transaminases, and risk of shock. As recovery progresses, reabsorption of extravasated fluid gradually restores hemodynamic stability; however, patients may experience reflex bradycardia (9–11).

Antibodies play dual roles in viral pathogenesis by mediating both protection and enhancement. While neutralizing antibodies clear viral particles, non-neutralizing or cross-reactive antibodies (12, 13) may induce ADE, immune complex deposition, and chronic inflammation (14, 15). In ADE, non-neutralizing antibodies form complexes with viruses, facilitating entry into Fc receptor-bearing cells (e.g., monocytes and macrophages), enhancing replication, immune hyperactivation, and tissue damage (15–18). In SARS-CoV-2 infection, immune-mediated tissue injury has been linked to complement activation and vasculitis (19, 20). Mechanisms contributing to pathogenic antibody responses include molecular mimicry, persistent viral antigenemia, and immune dysregulation with inappropriate cytokine signaling (21–25).

Cross-reactivity between DENV and SARS-CoV-2 has been previously reported. During the early pandemic, dengue seropositive results were observed in COVID-19 patients (26, 27), and anti-dengue antibodies were shown to bind to SARS-CoV-2 epitopes (28, 29). More recently, anti-SARS-CoV-2 antibodies targeting the spike S1 receptor-binding domain were demonstrated to recognize DENV antigens, including envelope (E), precursor membrane (prM), and non-structural protein 1 (NS1) (30). This structural homology may explain serological cross-reactivity and raise concerns regarding anti-SARS-CoV-2 antibodies triggering ADE in dengue, as suggested in recent reports (31–33).

After the COVID-19 pandemic, a marked increase in dengue incidence was reported in the Americas by World Health Organization (WHO)/Panamerican Health Organization (PAHO) (34). Post-acute sequelae of SARS-CoV-2 infection (long COVID) are characterized by persistent inflammation, autoimmunity, and vascular dysfunction, which are pathophysiological features shared with severe dengue (35, 36). These overlapping mechanisms raise the possibility that prior SARS-CoV-2 exposure may predispose individuals to enhanced dengue severity via shared pathways, such as complement activation, endothelial injury, and Th2-skewed immune responses.

Proteomic analysis is a powerful tool for elucidating host–pathogen interactions and identifying prognostic biomarkers of dengue infection (37–39). Previous studies have demonstrated that distinct proteomic signatures correlate with dengue severity and immune activation. Given the plausible role of anti-SARS-CoV-2 antibodies in modulating DENV infection, proteomics can illuminate the novel pathways and mediators underlying this interaction.

To investigate whether prior SARS-CoV-2 exposure modulates host response to acute dengue infection, we conducted a hypothesis-generating pilot study in a co-endemic Mexican population. Using label-free serum proteomic and *in vitro* ADE assays, we aimed to 1) characterize proteomic differences among patients stratified by dengue and SARS-CoV-2 serostatus, 2) identify differentially expressed proteins and enriched pathways linked to immune dysregulation, 3) assess antibody-mediated viral enhancement, and 4) identify candidate biomarkers and mechanistic pathways for further validation in larger prospective cohorts. This pilot investigation sought to generate preliminary evidence and testable hypotheses to inform future research on public health strategies in arbovirus-endemic regions affected by COVID-19.

## Materials and methods

This study adhered to the Strengthening the Reporting of Observational Studies in Epidemiology (STROBE) guidelines. A cross-sectional, observational pilot design was employed, enrolling 18 participants prospectively from Laboratorio Estatal de Salud Pública de Oaxaca, Mexico. The eligibility criteria were as follows: age 18–65 years, body mass index (BMI) <30 kg/m^2^, absence of chronic immunosuppressive conditions, no ongoing immunosuppressive therapy, and no history of malignancy, pregnancy, or recent vaccination (<2 weeks) against dengue or SARS-CoV-2. All the participants provided informed consent.

Participants were stratified into four cohorts: healthy controls (n = 3) and dengue-infected individuals (n = 15), divided by serological profiles. Dengue diagnosis required acute febrile illness (≥38 °C), characteristic clinical symptoms, and presentation within 7 days of symptom onset (actual range, 1–5 days). Controls were asymptomatic, seronegative for anti-SARS-CoV-2 and anti-dengue IgG, and had no recent infection.

Cohort classification integrates clinical diagnostics and serological testing. Dengue Duo rapid tests, ELISA-based assays for dengue NS1 antigen, anti-dengue IgG (Standard Q), RT-PCR (Ct < 40) for viral RNA, and anti-SARS-CoV-2 IgG ELISA (Euroimmun, Lübeck, Germany) were used. The resulting four groups were as follows: 1) CG (seronegative), 2) NS1/SARS_IgG (NS1+, anti-SARS-CoV-2 IgG+, and anti-dengue IgG−), 3) NS1/SARS-DENV_IgG (NS1+ and both antibodies+), and 4) NS1/DENV_IgG (NS1+, anti-dengue IgG+, and anti-SARS-CoV-2 IgG−).

Venous blood was collected in serum separator tubes, processed within 2 h, and stored at −80 °C. Pooled sera (≥300 µg protein per group) were used for proteomic analysis.

### Detection of antibodies

Anti-SARS-CoV-2 IgG was quantified using an ELISA kit (Euroimmun, Germany). Serum (1:100 dilution) was incubated in antigen-coated wells (60 min, 37 °C) followed by washing, enzyme-conjugate incubation (60 min, 37 °C), chromogenic substrate addition, and absorbance measurement at 450–630 nm using a chromate microplate reader (Awareness Technology Inc., Palm City, Florida, EE. UU). For anti-dengue IgG detection, we followed the Dengue Duo rapid test (SD Biosensor, Standard Q, Suwon, South Korea) protocol.

### RNA extraction and detection of dengue serotypes using qRT-PCR

Viral RNA was extracted from 140 μL of serum using a QIAamp Viral RNA Mini Kit (Qiagen, Venlo, Netherlands) with a final elution volume of 60 μL. RT-qPCR was performed using the Viasure Dengue Serotyping Real-Time PCR Kit. Amplification was performed using a Fast ABI 7500 thermocycler with the following parameters: 45 °C for 15 min (reverse transcription), 95 °C for 2 min (denaturation), and 45 cycles of 95 °C for 10 s, and 60 °C for 50 s. A Ct value <40 was considered positive.

### Protein integrity assessment

Protein integrity was assessed using 12% Sodium Dodecyl Sulfate-Polyacrylamide Gel Electrophoresis (SDS–PAGE). Serum samples (5 µg protein) were mixed with Laemmli buffer, resolved by electrophoresis (90 min at 110 V) using a Mini-PROTEAN system (Bio-Rad, Hercules, California), and stained with 0.02% Coomassie Brilliant Blue G-250. Samples showing the expected human serum protein bands (≥10 kDa) were selected for further analysis.

### Inflammatory marker quantification

IL-6 and C-reactive protein (CRP) levels were quantified using Finecare immunofluorescence assays (catalog no. W251 and W201, Wondfo, Guangzhou, China), according to the manufacturer’s instructions. Fluorescence was detected using the Finecare FIA Meter II Plus instrument.

### Label-free quantitative proteomic analysis of serum proteome

Following group classification, serum samples from each cohort were pooled before proteomic analysis to minimize individual-level variability and address potential confounding factors in the analysis. Label-free quantitative bottom-up proteomics was performed on pooled samples separated by gel electrophoresis, followed by band excision and in-gel trypsin digestion (Promega, Madison, WI, USA). The peptides were resuspended in 20 μL of 0.1% formic acid prior to LC–MS/MS. Proteomic analyses were conducted by Creative Proteomics (Shirley, NY, USA) using an Ultimate 3000 nano UHPLC system (Thermo Fisher Scientific, Waltham, USA) with an Electrospray Ionization (ESI) nanospray source, incorporating a trapping column (PepMap C18, 100 Å, 100 μm × 2 cm, 5 μm) and an analytical column (PepMap C18, 100 Å, 75 μm × 50 cm, 2 μm). The sample loading was 1 µg. The mobile phase consisted of 0.1% formic acid in water (A) and 0.1% formic acid in 80% acetonitrile (B) (v/v) at a flow rate of 250 nL/min. The LC gradient was as follows: 2%–8% buffer B for 3 min, 8%–20% for 56 min, 20%–40% for 37 min, and 40%–90% for 4 min. Mass spectrometric analysis included a scan across the 300–1,650 m/z range with 60,000 resolution at 200 m/z. The full-scan automatic gain control target was 3e6 ions. MS/MS scan used Top 20 mode with 15,000 resolution, 1e5 automatic gain control, 19-ms injection time, 28% collision energy, and 1.4 Th isolation window. Charge-state exclusion was applied to unassigned, 1, and >6 states, with 30-s dynamic exclusion.

### Data analysis

Four raw MS files were analyzed against a human protein database using MaxQuant (1.6.2.6). The parameters included carbamidomethylation (C) (fixed) and oxidation (M) (variable) modifications, trypsin enzyme specificity, maximum missed cleavage of 2, precursor ion mass tolerance of 10 ppm, and MS/MS tolerance of 0.5 Da. A four-way Venn diagram was created to identify differentially expressed proteins (DEPs) using interactiVenn (http://www.interactivenn.net/, accessed on 29 January 2025). Protein abundance was quantified using the ratio of abundance (RA), normalizing protein levels in dengue patients to those in healthy controls. Proteins with RA > 1 were classified as upregulated proteins. A heatmap was generated via Morpheus (https://software.broadinstitute.org/morpheus; accessed 29 January 2025), stratifying proteins with RA > 1.0 and RA < 1.0 across groups. Hierarchical clustering delineated the group-specific proteomic signatures. Gene enrichment analysis was performed using ShinyGO 0.80 (Ge SX, Jung D & Yao R, Bioinformatics 36:2628–2629, 2020).

### Cell culture

Vero (ATCC CCL-81), K562 (ATCC CCL-243), and BHK-21 (ATCC CCL-10) cell lines were cultured in Roswell Park Memorial Institute (RPMI) or Dulbecco's Modified Eagle Medium (DMEM) supplemented with 3%–5% Fetal Bovine Serum (FBS), l-glutamine, antibiotics, and 10 mM NaHCO_3_, and incubated at 37 °C in 5% CO_2_.

### Viral propagation

DENV-1 (Western Pacific strain, GenBank accession number AY145121.1) was propagated in Vero cells. High-titer viral stocks were stored at −70 °C until use.

### Infection and ADE assays

To assess ADE, heat-inactivated pooled sera were heat-inactivated (56 °C, 30 min) and incubated with DENV-1 (Multiplicity of infection (MOI) 0.5, 1 × 10^5^ PFU) at 37 °C for 1 h. The mixtures were added to K562 cells (2 × 10^5^ cells/well, 24-well plates) in triplicate. The virus-only and Phosphate Buffered Saline (PBS) control groups were also included. After 24 h, the supernatants were collected for virus titration.

### Plaque assays for viral quantification

BHK-21 cells were seeded in 24-well plates at 5 × 10^4^ cells/well for 24 h. Supernatants collected at 24 and 48 h were diluted 10-fold (1:10 to 1:10^6^) in 96-well plates. Each dilution (100 µL) was added to 24-well plates and incubated at 37 °C in 5% CO_2_ for 1 h. Monolayers were overlaid with a semisolid medium (RPMI, 2% FBS, penicillin/streptomycin, and 1.25% methylcellulose) and incubated at 37 °C for 4 days. The plates were PBS-washed, fixed with paraformaldehyde, stained with crystal violet, rinsed, and counted for clear areas. Viral titers were expressed as log_10_ PFU/mL.

## Results

### Cohort characteristics and clinical profiles

Participants were stratified into four cohorts: healthy controls (CG; n = 3), dengue-infected patients with anti-SARS-CoV-2 IgG antibodies (NS1/SARS_IgG; n = 5), dengue-infected patients with both anti-SARS-CoV-2 and anti-dengue IgG antibodies (NS1/SARS-DENV_IgG; n = 5), and dengue-infected patients with anti-dengue IgG antibodies (NS1/DENV_IgG; n = 5). The complete clinical and laboratory profiles are summarized in **Table 1**.

**Table 1:** Descriptive clinical and laboratory characteristics of the study participants stratified by serological status.

Symptoms	Control n = 3	NS1/SARS_IgG n = 5	NS1/SARS-DENV_IgG n = 5	NS1/DENV_IgG n = 5
Fever (°C, $\bar{\text{x}}$)	–	38.6 ± 0.54	38.3 ± 0.51	39 ± 0
Headache (%)	–	100	100	100
Myalgia (%)	–	100	83.3	100
Arthralgia (%)	–	100	83.3	100
Retro-orbital pain (%)	–	60	50	75
Abnormal hematocrit (%)	–	20	0	25
Thrombocytopenia (%)	–	20	16.6	50
Nausea (%)	–	40	16.6	50
Vomiting (%)	–	20	0	50
Serological status				
Dengue NS1	Negative	Positive	Positive	Positive
Anti-SARS-CoV-2 IgG	Negative	Positive	Positive	Negative
Anti-Dengue IgG	Negative	Negative	Positive	Positive
Proinflammatory tests*				
IL-6 (pg/mL)	<3.0	18.3 ± 15.6	4.5 ± 1.2	<3.0
CRP (mg/L)	<5.0	51.6 ± 29.6	38.3 ± 19.1	<5.0

CRP, C-reactive protein.

*Data are presented as mean ± SD or percentages for descriptive purposes. Statistical significance testing was not performed due to the small sample size (n = 5 per group). These findings are descriptive observations in a pilot cohort and should not be interpreted as statistically significant group differences.

All dengue-infected groups exhibited 100% incidence of headache. The NS1/DENV_IgG group presented with the most severe clinical phenotype, marked by elevated fever (39 °C), retro-orbital pain (75%), thrombocytopenia (50%), nausea (50%), vomiting (50%), and abnormal hematocrit (25%). Myalgia and arthralgia were observed in all patients in the NS1/SARS_IgG and NS1/DENV_IgG groups, but with a lower incidence (83.3%) in the NS1/SARS-DENV_IgG group. Retro-orbital pain was more frequent in the NS1/DENV_IgG group (75%) than in the NS1/SARS_IgG (60%) and NS1/SARS-DENV_IgG (50%) groups. Notably, thrombocytopenia and vomiting were absent in the NS1/SARS-DENV_IgG group, which also had the lowest incidence of nausea (16.6%) and no cases of abnormal hematocrit (**Table 1**).

Inflammatory markers corroborated these trends, IL-6 levels peaked in the NS1/SARS_IgG group (18.3 ± 15.6 pg/mL), followed by the NS1/SARS-DENV_IgG group (4.5 ± 1.2 pg/mL). Both the CG and NS1/DENV_IgG groups had undetectable IL-6 (<3.0 pg/mL). CRP levels were likewise elevated in the NS1/SARS_IgG group (51.6 ± 29.6 mg/L) and NS1/SARS-DENV_IgG (38.3 ± 19.1 mg/L), but were below the detection limits in the CG and NS1/DENV_IgG groups (<5.0 mg/L). These patterns suggest a heightened systemic inflammation associated with anti-SARS-CoV-2 IgG seropositivity (**Table 1**).

### Proteomic profiling and differential expression

**Figure 1:**
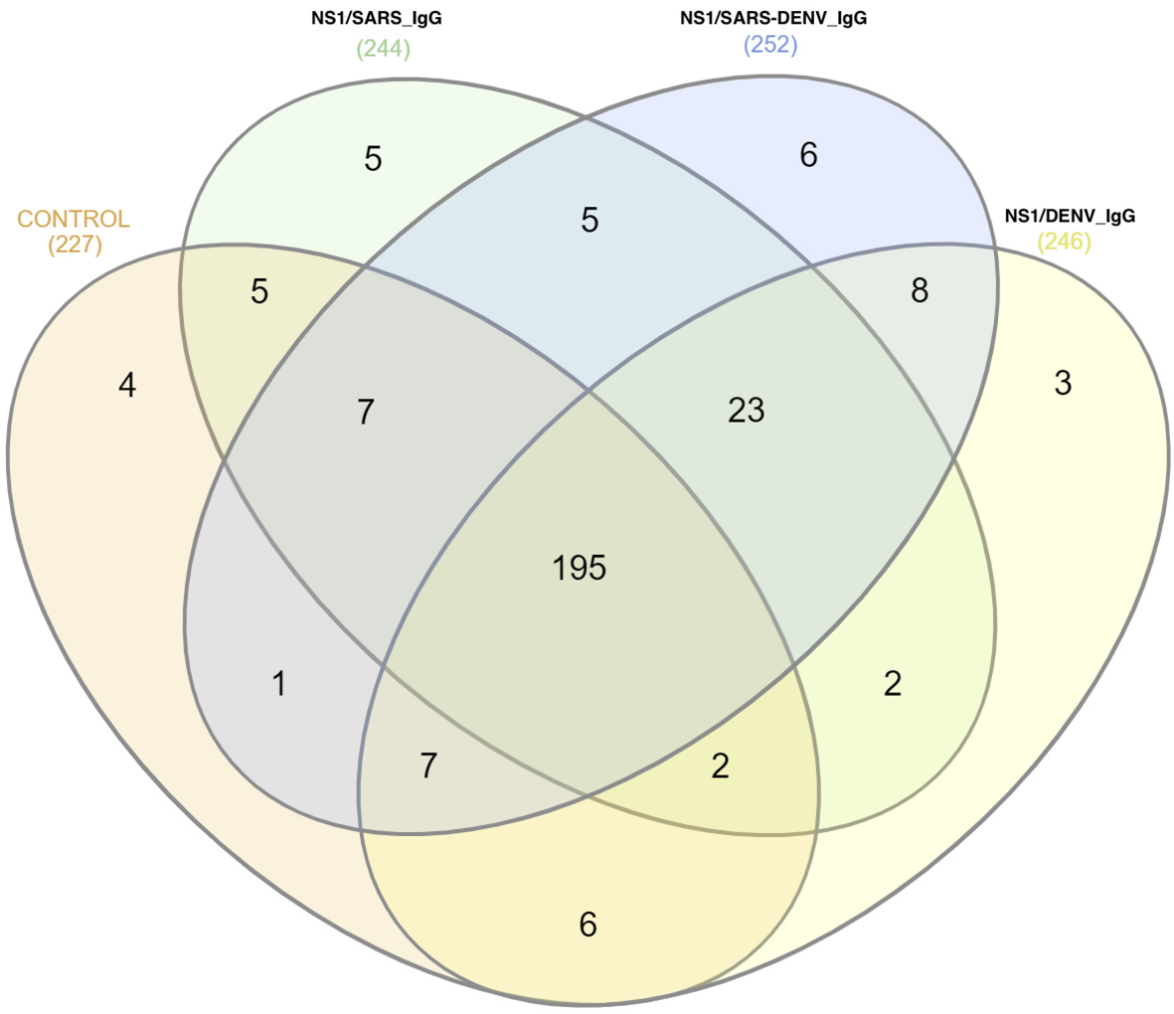
Differentially expressed proteins and pathway dysregulation across serological cohorts. Four-way Venn diagram displaying the distribution of 279 identified proteins across study groups, with 18 differentially expressed proteins (DEPs) identified as cohort-specific. KEGG pathway enrichment analysis reveals significant dysregulation of complement/coagulation cascades (p = 0.016) and TGF-β signaling (p = 0.022) in the NS1/SARS-DENV_IgG group. Gene Ontology analysis indicates Th2-polarized immune responses (immune response to nematodes, p = 0.001; negative regulation of cytokine production, p = 0.003) associated with cross-reactive antibody-mediated immune dysregulation.

High-confidence proteomic profiling identified 1,121 unique sequences, of which 279 were retained after filtering (non-gene-associated entries, reverse matches, uncharacterized proteins, duplicates, and sex-specific proteins). Group-specific protein distribution revealed the following: CG (227 proteins), NS1/SARS_IgG (244), NS1/SARS-DENV_IgG (252), and NS1/DENV_IgG (246) (**Figure 1**). Kyoto Encyclopedia of Genes and Genomes (KEGG) enrichment analysis highlighted neutrophil extracellular trap (NET) formation and Peroxisome Proliferator-Activated Receptor Signaling Pathway (PPAR) signaling in the infected cohorts. Notably, the phagosome pathway was exclusive to NS1/SARS-DENV_IgG.

**Figure 2:**
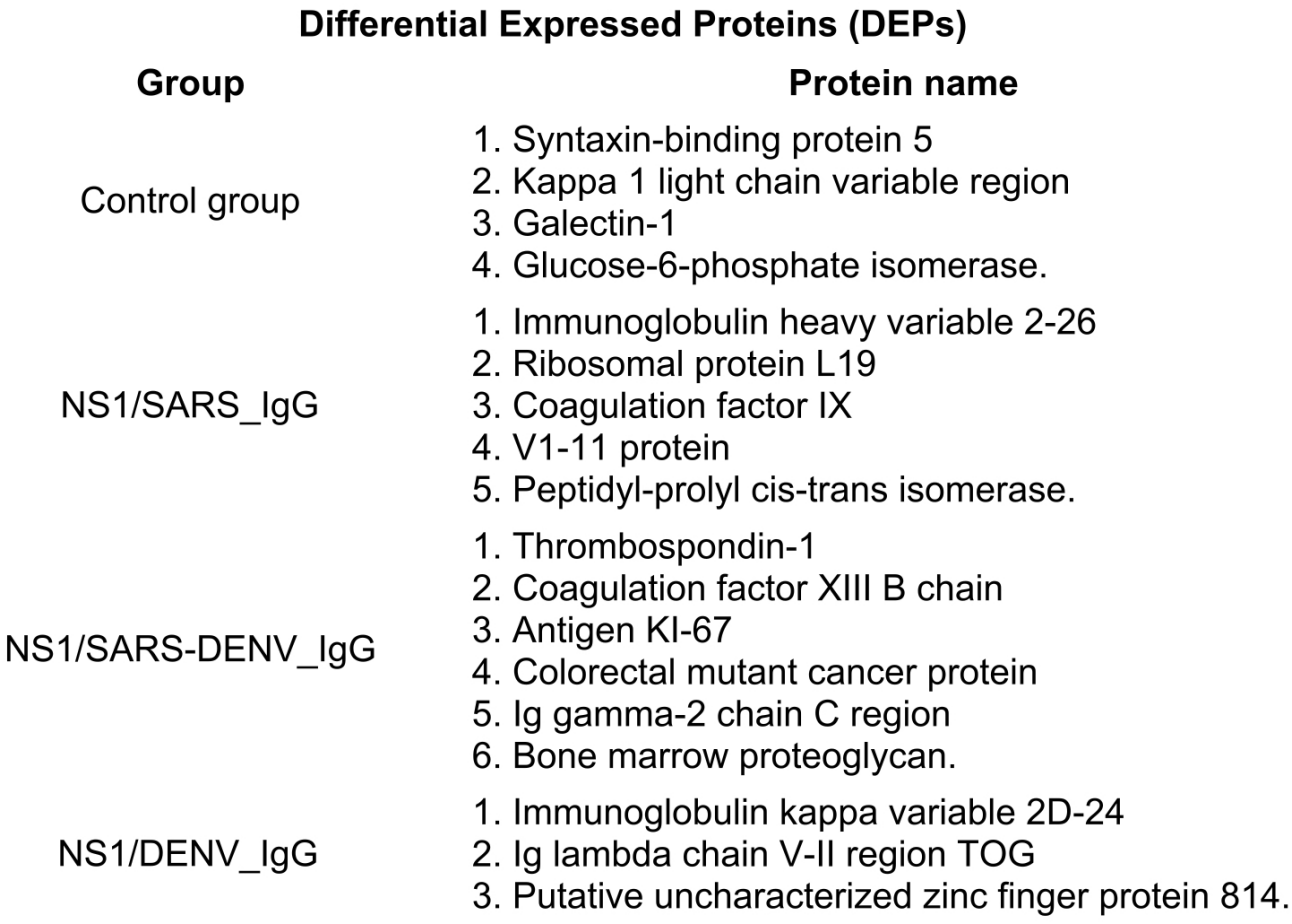
Differentially expressed proteins (DEPs) stratified by study cohort. Eighteen DEPs identified across four study groups reveal cohort-specific proteomic signatures. Control group (n = 4 DEPs): baseline serum proteins including STXBP5, kappa-1 light chain, galectin-1, and GPI. NS1/SARS_IgG (n = 5 DEPs): immunoglobulin heavy chain variable 2-26, ribosomal protein L19, coagulation factor IX, V1–11 protein, and PPIB (indicating enhanced host replication factors and coagulation activation). NS1/SARS-DENV_IgG (n = 6 DEPs): thrombospondin-1, coagulation factor XIII B chain, antigen KI-67, colorectal mutant cancer protein, Ig gamma-2 chain C region, and bone marrow proteoglycan (suggesting platelet activation, proliferation, and Th2-polarized immunity). NS1/DENV_IgG (n = 3 DEPs): immunoglobulin kappa variable 2D-24, Ig lambda chain V-II region TOG, and putative zinc finger protein 814. GPI, glucose-6-phosphate isomerase; PPIB, peptidyl-prolyl *cis*–*trans* isomerase B.

DEPs (n = 18) displayed distinct cohort-specific patterns: CG (n = 4), NS1/SARS_IgG (n = 5), NS1/SARS-DENV_IgG (n = 6), and NS1/DENV_IgG (n = 3). Functional annotation indicated that most DEPs were associated with coagulation cascades or antiviral responses. The rheumatoid factor C6 light chain, immunoglobulin variants (heavy variable 2–26, V1–11; kappa variable 2D-24, V1–4), and putative zinc finger protein 814 have no well-established roles in viral pathogenesis. Proteins, such as thrombospondin-1 and STXBP5, were enriched in NS1/SARS-DENV_IgG, suggesting Th2-skewed responses and enhanced platelet activation. Other DEPs included metabolic (glucose-6-phosphate isomerase), immune-regulatory (syntaxin-binding protein 5), and potential viral entry mediators (galectin-1) (**Figure 2**).

**Figure 3:**
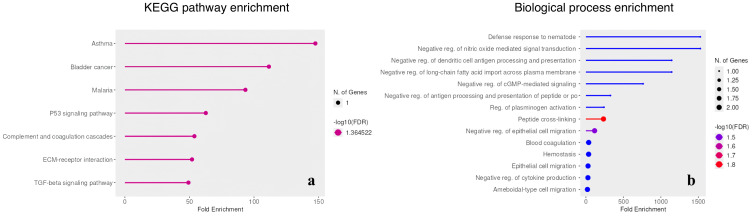
Pathway and biological process enrichment analysis of the NS1/SARS-DENV_IgG group. **(a)** KEGG pathway enrichment: bubble plot showing significant dysregulation of pathways associated with severe dengue pathogenesis, specifically complement and coagulation cascades (p = 0.016, fold enrichment ~55) and TGF-β signaling (p = 0.022, fold enrichment ~50). These pathways indicate immune and vascular dysregulation characteristic of severe disease. **(b)** Biological process enrichment: analysis reveals a distinct Th2-polarized immune signature, highlighted by enrichment in immune response to nematodes (GO:0052176, p = 0.001) and negative regulation of cytokine production (GO:0001818, p = 0.003). Additional enrichment in eosinophil activation pathways supports a shift toward type 2 immunity, potentially facilitating viral enhancement rather than neutralization. Bubble size represents gene count; color intensity indicates statistical significance (-log10 (False Discovery Rate (FDR)))..

The pathway enrichment analysis of the NS1/SARS-DENV_IgG group revealed significant associations with severe dengue pathogenesis, including complement and coagulation cascades (p = 0.016) and TGF-β signaling (p = 0.022) (**Figure 3a**). Biological process analysis further revealed Th2-polarized immune mechanisms, including the immune response to nematodes (GO:0052176, p = 0.001) and negative regulation of cytokine production (GO:0001818, p = 0.003), indicating negative regulation of cytokine production and Th2-associated responses, including eosinophil activation pathways (**Figure 3b**).

Abundance ratio (AR) analysis relative to CG identified 95 upregulated proteins (AR > 1.0) and 132 downregulated proteins (AR < 1.0) in the NS1/SARS_IgG group, the NS1/SARS-DENV_IgG group had 86 upregulated and 141 downregulated proteins, and the NS1/DENV_IgG group had 92 upregulated and 135 downregulated proteins (**Supplementary Figure S1**). Enrichment analysis of AR > 1.0 proteins showed immune perturbations specific to each group in biological processes: enrichment in B cell-mediated immunity, immune activation (including lymphocyte and leukocyte effector processes), and inflammatory responses, including pathogen defense mechanisms in the NS1/SARS_IgG group, dysregulation of complement activation (alternative pathway), inhibition of peptidase/protease activity (e.g., negative regulation of endopeptidases), modulation of proteolytic cascades (NS1/SARS-DENV_IgG), enrichment in coagulation homeostasis (hemostasis and blood clot regulation), wound healing, and systemic responses to external stimuli (NS1/DENV_IgG).

**Figure 4:**
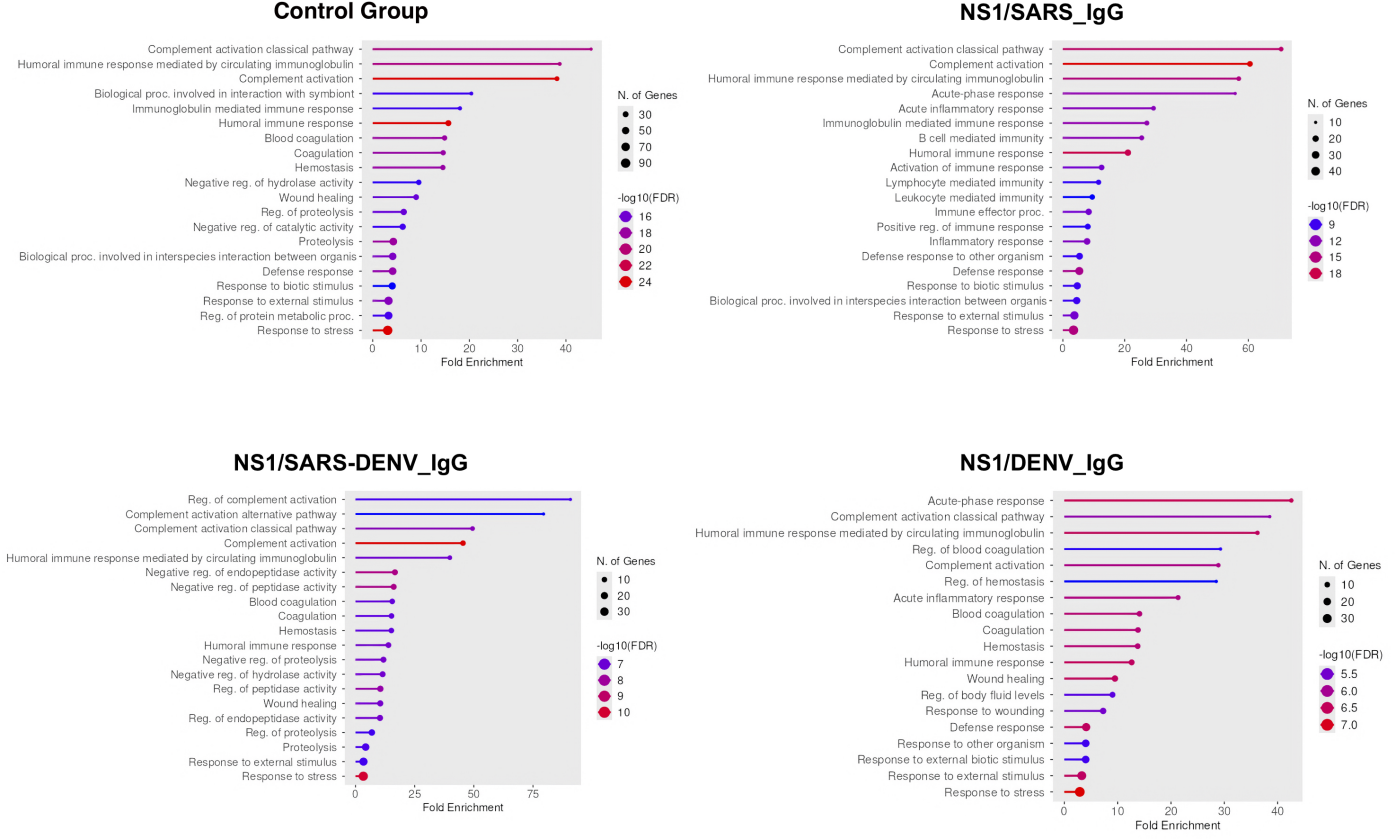
Gene Ontology cellular component enrichment analysis of proteins with AR > 1.0 across study cohorts. Four-panel bubble plot showing cellular component enrichment by serological group. Control group demonstrates baseline lipoproteins and platelet granules. NS1/SARS_IgG exhibits highest enrichment in membrane attack complex and pore complex (fold enrichment ~200), indicating complement dysregulation. NS1/SARS-DENV_IgG shows moderate enrichment in membrane and granule components (fold enrichment ~150). NS1/DENV_IgG reveals plasma lipoprotein and platelet granule enrichment (fold enrichment ~40). Bubble size represents gene number (10–125); color intensity reflects significance [−log10(FDR), 10–30]. AR, abundance ratio.

Cellular component analysis revealed platelet alpha granule enrichment across infected groups, with pore complex structures enriched in the NS1/SARS_IgG group, and vacuolar lumen components uniquely characterized in the NS1/SARS-DENV_IgG group (**Figure 4**).

**Figure 5:**
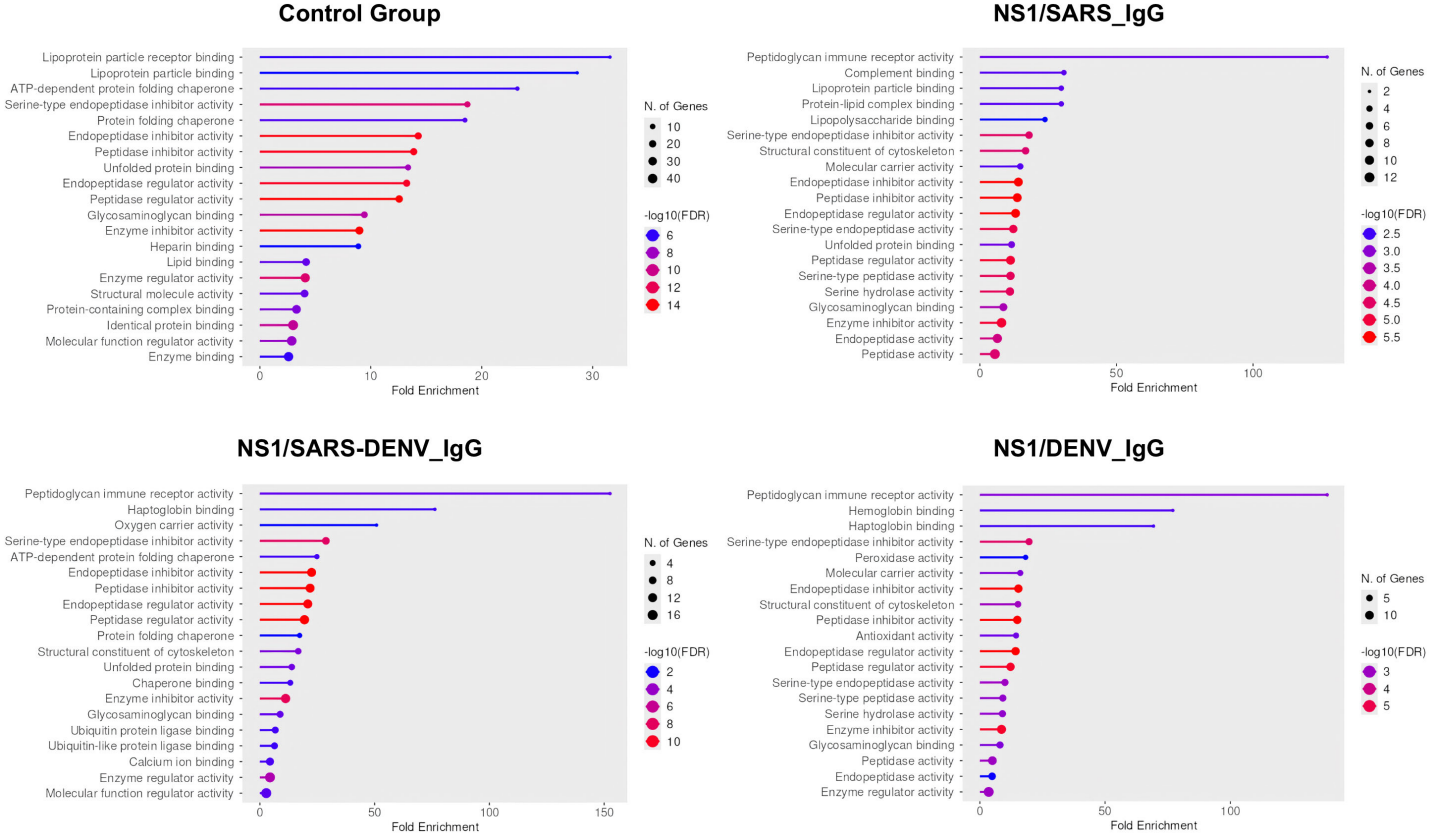
Gene Ontology molecular function enrichment analysis of proteins with AR > 1.0 across study cohorts. Four-panel bubble plot displaying molecular function enrichment by serological status. Control group shows lipoprotein particle receptor binding (fold enrichment ~30). NS1/SARS_IgG exhibits highest peptidoglycan immune receptor activity enrichment (fold enrichment ~120), indicating immune activation. NS1/SARS-DENV_IgG demonstrates peptidoglycan immune receptor and haptoglobin binding enrichment (fold enrichment ~100–140). NS1/DENV_IgG reveals dominant peptidoglycan immune receptor activity (fold enrichment ~100). Bubble size indicates gene number (2–18); color intensity represents significance [−log10(FDR), 2.5–10].

Molecular function analysis revealed functions associated with peptidoglycan immune receptor activity, structural constituents of the cytoskeleton, molecular carrier activity, serine-type endopeptidase activity, serine-type peptidase activity, serine hydrolase activity, endopeptidase activity, and haptoglobin binding in infected cohorts. Additional functions related to complement, protein–lipid complexes, and lipopolysaccharide binding were found exclusively in the NS1/SARS_IgG group. Oxygen carrier activity, chaperone binding, ubiquitin protein ligase binding, ubiquitin-like protein ligase binding, and calcium ion binding were observed in the NS1/SARS-DENV_IgG group, whereas hemoglobin binding, peroxidase activity, antioxidant activity, and peptidase activity were observed in the NS1/DENV_IgG group (**Figure 5**).

### *In vitro* ADE assays

**Figure 6:**
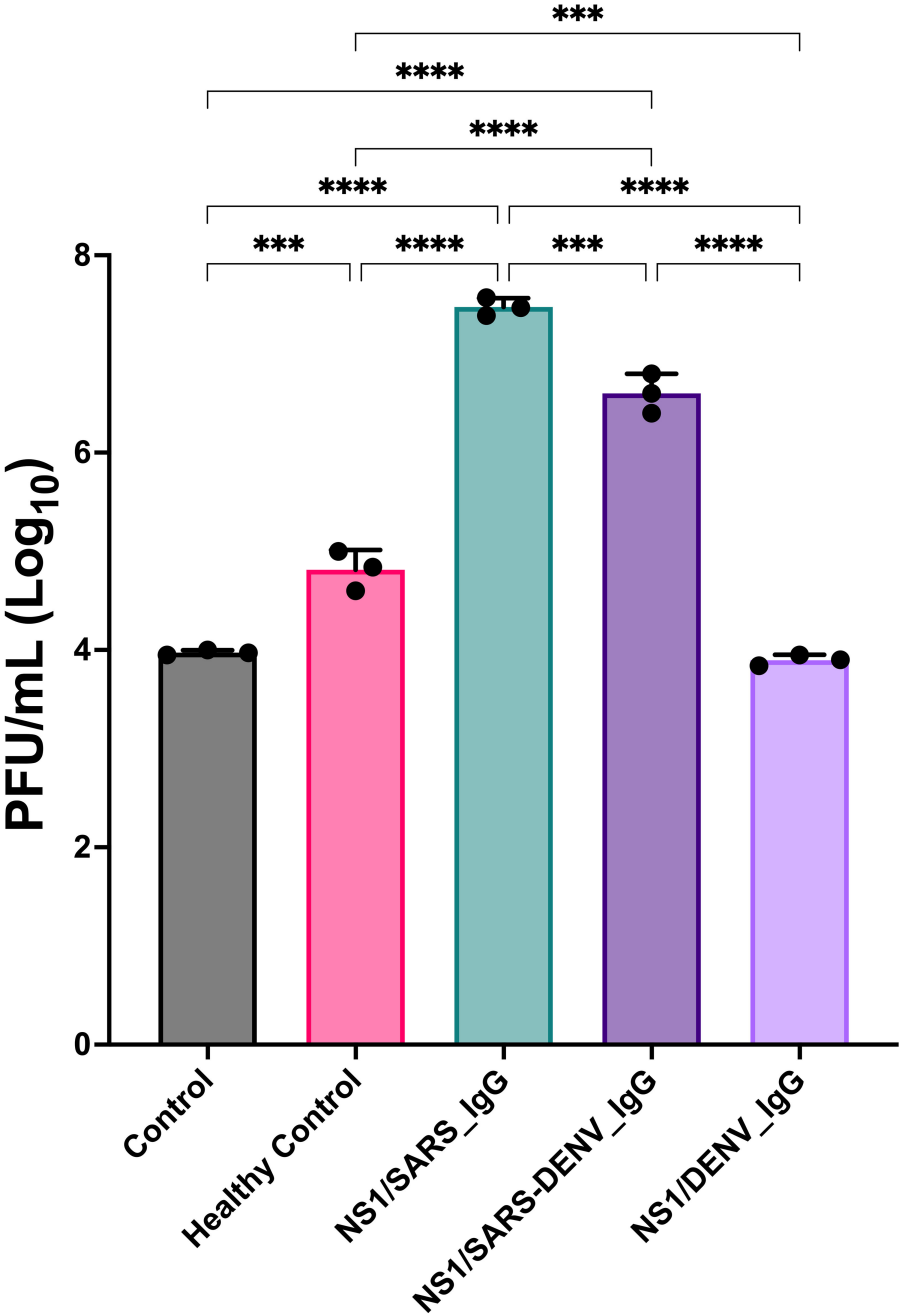
Viral titers 1 day post-infection in K562 cells under different conditions with DENV and antibodies. Data are presented as median and standard deviation of three independent replicates. Statistical analysis was performed using one-way ANOVA and Dunnett’s *post-hoc* test to compare conditions with the control (Ctrl); *** means p < 0.001; *** means p < 0.001; **** indicates p < 0.0001. DENV, dengue virus.

DENV-1 infection of K562 cells in the presence of pooled sera revealed enhanced viral replication in groups treated with anti-SARS-CoV-2 IgG. The NS1/SARS_IgG group exhibited the highest titers, followed by the NS1/SARS-DENV_IgG. NS1/DENV_IgG sera showed titers comparable to those of the virus-only controls, indicating minimal enhancement. These results confirm that anti-SARS-CoV-2 antibodies facilitate ADE *in vitro*, which is consistent with the clinical and proteomic observations (**Figure 6**).

## Discussion

This pilot study revealed similar clinical manifestations among groups, as expected during the acute phase of dengue, reinforcing the challenge of severity prediction in the early infection stages. The proinflammatory markers IL-6 and CRP were elevated in patients with anti-SARS-CoV-2 and dengue IgG antibodies compared with those with only anti-dengue IgG antibodies, highlighting the potential role of anti-SARS-CoV-2 antibodies as cross-reactive antibodies. IL-6 and IL-10 levels have been associated with secondary infections and ADE (40), suggesting a plausible mechanism of pathogenic crosstalk via molecular mimicry between SARS-CoV-2 spike epitopes and dengue E proteins. Unfortunately, our sample size limits this conclusion; therefore, further analysis is needed to clarify this point.

Elevated IL-6 and CRP in the groups with SARS_IgG suggest that cross-reactive anti-SARS-CoV-2 antibodies facilitate viral replication, amplifying inflammation, potentially via alternative ADE pathways involving immune complex-mediated activation and complement deposition (11, 41, 42). This could explain the enhanced thromboinflammatory proteome in the NS1/SARS-DENV_IgG group despite lower inflammation markers, indicating distinct pathogenic trajectories: classical ADE-dominated replication in the NS1/SARS_IgG group versus a hybrid of enhanced infection and autoantibody-driven damage in the NS1/SARS-DENV_IgG group, consistent with the two-hit model described by Sun et al. (2025) (41).

Proteomic analysis identified 1,121 protein sequences, of which 279 were mapped to known protein-coding genes, revealing significant proteome remodeling during infection, with distinct expression patterns across cohorts. Patients with anti-SARS-CoV-2 or dengue antibodies exhibited similar protein number, but dual-seropositive individuals (SARS-CoV-2 and dengue IgG) exhibited the highest protein diversity, surpassing any other group and potentially linked to a worsened clinical outcome (38, 39). The phagosome pathway exclusive to the NS1/SARS-DENV_IgG group suggests early opsonization facilitated by dual antibodies, while NET formation and PPAR signaling across infected groups emphasize proteomic confidence in uncovering infection-specific events (43, 44).

The proteomic signatures identified in this study were contextually grounded in cohorts stratified by serological status and acute infection phase. The narrow temporal enrollment window (1–5 days post-symptom onset) ensured that the observed protein abundance differences reflected acute immune responses specific to the infection stage and prior viral exposure, rather than confounding from disease progression. Group-specific pathway enrichment (complement/coagulation cascades in NS1/SARS-DENV_IgG; elevated acute-phase response in NS1/SARS_IgG) aligns with the known dengue pathophysiology and ADE mechanisms, strengthening biological plausibility.

The clinical consistency within groups (**Table 1**), including fever, symptom prevalence, and inflammatory markers, validated that the identified proteomic changes corresponded to meaningful immunological phenotypes rather than noise or confounding. For example, the NS1/SARS-DENV_IgG group’s enrichment in complement/coagulation pathways (p = 0.016) and TGF-β signaling (p = 0.022) corresponded clinically to their milder thrombocytopenia (16.6%) and absence of hematocrit abnormalities, consistent with a Th2-skewed, non-hemorrhagic phenotype. Conversely, the NS1/DENV_IgG group’s highest fever (39 °C) and highest thrombocytopenia (50%) correlated with their distinct proteomic profile, supporting the hypothesis that serological status modulates immune and coagulation responses.

Analysis of DEPs revealed group-specific implications for dengue pathogenesis. Healthy controls: Galectin-1 (LGALS1), a lectin with antiviral activity via viral replication suppression in murine *in vitro* models (45), was undetectable in the infected cohorts, potentially enabling unchecked viral propagation. Syntaxin-binding protein 5 (STXBP5), expressed in endothelial cells and platelets, has emerged as a candidate; its deficiency elevates P-selectin and von Willebrand factor (vWF) (46), promoting pathological platelet adherence (47), a hallmark of severe dengue. STXBP5 knockout in mice reduces platelet factor 4 (PF4/CXCL4) secretion (48), mirroring the observed PF4 reduction, which correlates with dengue severity (49, 50). The absence of glucose-6-phosphate isomerase (GPI) suggests host metabolic restriction, impeding the glycolytic flux exploited by viruses via the Warburg effect (51). These shifts highlight novel host–pathogen interactions that require further study.

In the NS1/SARS_IgG group, DEPs such as ribosomal protein L19 (RPL19) and peptidyl-prolyl *cis*–*trans* isomerase B (PPIB) have been implicated as host factors for flavivirus replication (52, 53). Coagulation factor IX detection reflects ongoing coagulopathy, which is consistent with dengue-induced platelet aggregation, endothelial tissue factor expression, and coagulation cascade activation (54, 55). Enrichment in complement activation, COVID-19, and ribosome pathways suggests sustained inflammation, aligning with thromboinflammatory and proliferative signatures (56). Elevated IL-6/CRP corroborates the coagulation–proinflammatory interplay in the immune response in dengue pathogenesis.

The dual seropositivity for the SARS-CoV-2 and dengue antibody groups exhibited the most DEPs. Thrombospondin-1 (THBS1), released from α-granules of activated platelets, points to aggregation and consumption, correlating with thrombocytopenia, bleeding, plasma leakage, and organ failure in severe dengue (57), peaking critically (days 3–4 post-infection) and resolving post-convalescence (58). Premature acute activation may deplete platelets, thereby increasing hemorrhage risk (59). Coagulation factor XIII B chain (FIIIB) suggests a potential coagulation pathway dysfunction in FXIII–A2B2 complex formation. Bone marrow proteoglycan (PRG2) from activated eosinophils induces platelet activation via IL-4 and IL-10 during Th2 polarization (60). Elevated IgE levels in severe dengue and ADE underscore the understudied role of eosinophils (61). Th2 skewing, an ADE hallmark, suppresses antiviral IFN-γ responses while promoting immunopathological changes. Enrichment analysis revealed that KEGG pathways are associated with complement/coagulation cascades and TGF-β signaling, both associated with severe dengue (62).

The antigen KI-67 (MKI67) and colorectal mutant cancer protein (MCC), which are proteins that regulate cell proliferation, were uniquely upregulated in the NS1/SARS-DENV_IgG group. While their roles in dengue remain uncharacterized, MKI67’s association with proliferating cells (e.g., plasmablasts) and MCC’s NF-κB modulation by MCC suggest a potential involvement in viral replication or immune dysregulation. Further studies should explore whether these proteins represent host defense mechanisms or viral exploitation strategies.

NS1/SARS-DENV_IgG group’s proteomic profile reflects synergistic immune dysregulation; cross-reactive antibodies (anti-dengue/anti-SARS-CoV-2) may enhance complement activation and Th2 polarization, which is an inefficient antiviral response. These findings highlight the clinical urgency of monitoring dengue severity in SARS-CoV-2-exposed populations, particularly in co-endemic regions. Future studies should validate these mechanisms in longitudinal cohorts and explore therapeutic strategies targeting platelet activation, coagulation imbalances, and Th2 cytokines to mitigate severe outcomes.

Although DEPs in the NS1/DENV_IgG group lacked direct functional annotations, the identification of putative zinc finger protein 814 (ZNF814)—a zinc finger family member implicated in the transcriptional regulation of antiviral immunity—suggests potential roles in immune modulation during dengue infection. Zinc finger proteins regulate host–pathogen interactions through chromatin remodeling and RNA-binding activities during viral restriction or immune evasion (63). Further characterization of ZNF814, including its interaction with dengue viral components or host signaling pathways (e.g., IFN response), is critical to elucidate its contribution to disease progression.

The identified DEPs, such as STXBP5, THBS1, and PRG2, have potential as biomarkers for predicting severe dengue outcomes. Their roles in platelet activation, coagulation dysfunction, and Th2 immune polarization suggest their utility in stratifying patients at risk of severe disease. However, validation using larger cohorts and clinical trials is essential before clinical implementation.

Comparative analysis of protein abundance ratios (AR > 1.0) identified 227 dysregulated proteins between healthy donors and dengue-infected patients. Among the infected cohorts, the NS1/SARS_IgG and NS1/DENV_IgG groups exhibited similar upregulated protein counts (95 and 92, respectively), while the dual-seropositive group (NS1/SARS-DENV_IgG) showed reduced diversity (86 proteins), suggesting distinct immune adaptations potentially driven by cross-reactive antibody influences. Notably, proteins associated with dengue severity, such as C1q and C3 from the complement system, CRP as an acute-phase reactant (64, 65), indicators of hypoalbuminemia (66), and downregulation of apolipoproteins (APOA1 and APOB) (67) were consistently altered across the infected groups, particularly in the NS1/SARS_IgG and NS1/DENV_IgG groups. These alterations align with prior reports of complement activation and lipid dysregulation in severe dengue and extend to parallels with virus-induced pathogenic antibodies in long COVID and Dengue Hemorrhagic Fever (DHF), where immune complexes and autoantibodies exacerbate inflammation and vascular damage. In the context of the two-hit model for dengue severity, these proteomic shifts could represent the “first hit” of viremia-induced inflammation (e.g., acute-phase CRP elevation), primed by cross-reactive anti-SARS-CoV-2 antibodies in dual exposures, setting the stage for a “second hit” of autoantibody-mediated damage during the critical phase (41).

Enrichment analysis of AR > 1.0 proteins highlighted acute immune activation pathways in all the infected groups. However, the NS1/SARS_IgG group uniquely exhibited leukocyte- and lymphocyte-mediated responses, alongside complement activation, indicative of heightened inflammation, potentially amplified by molecular mimicry between SARS-CoV-2 and DENV infection. Interestingly, only the NS1/SARS-DENV_IgG group demonstrated enrichment of the alternative complement pathway, a recognized driver of severe dengue pathogenesis (65), similar to immune complex formation in the persistent dysregulation of long COVID. This group-specific pattern suggests that dual seropositivity shifts dengue toward a “hybrid” phenotype, blending DHF’s acute vascular permeability with long COVID’s chronic autoimmunity, where Th2-skewed responses and sustained inflammation bridge these conditions.

Cellular component analysis revealed platelet α-granule enrichment in the infected cohorts, consistent with dengue-associated platelet activation and consumptive coagulopathy, a hallmark of DHF exacerbated by pathogenic antibodies. Molecular function profiling revealed the absence of lipoprotein particle receptor binding, consistent with dengue virus-induced lipoprotein dysregulation and lipid metabolism suppression (68). This aligns with the viral exploitation of host lipids for replication and immune evasion, further supporting the role of persistent viral antigens in driving abnormal antibody production and prolonged inflammation, as seen in both long COVID and secondary DENV infections.

Infection assays revealed that K562 cells infected with DENV-1 in the presence of serum containing anti-SARS-CoV-2 antibodies exhibited heightened permissiveness for viral replication, with the NS1/SARS_IgG group showing the highest titers, followed by the NS1/SARS-DENV_IgG group, while NS1/DENV_IgG displayed the lowest, comparable to the virus-only control (**Figure 6**). Serum from healthy donors permitted intermediate replication, exceeding NS1/DENV_IgG but remaining below other groups. This pattern aligns with classical ADE mechanisms, in which non-neutralizing or sub-neutralizing antibodies facilitate viral entry into Fc receptor-bearing cells, enhancing viral replication and disease severity (41). Cross-reactive anti-SARS-CoV-2 antibodies, likely targeting DENV epitopes such as E, PrM, or NS1 (33, 69), may form immune complexes during pre-incubation, promoting this enhancement effect. Patient-derived sera from Groups 1 and 2, potentially containing pre-existing virus–antibody complexes, could further amplify replication. In contrast, anti-dengue antibodies in the NS1/SARS-DENV_IgG and NS1/DENV_IgG groups appeared to exert a neutralizing effect, mitigating ADE and reducing titers in the NS1/DENV_IgG group, with partial attenuation in the NS1/SARS-DENV_IgG group due to competing cross-reactive influences. Our results align with recent findings by Reinig et al. (33), demonstrating that COVID-19 vaccination induces cross-reactive dengue antibodies with altered isotypes that facilitate *in vitro* ADE, supporting the notion that anti-SARS-CoV-2 antibodies cross-react with DENV and drive replication similar to non-specific or enhancing antibodies (6, 70, 71). Future studies should systematically evaluate serotypes 2, 3, and 4 to determine whether cross-reactive ADEs are serotype-specific or broadly applicable. However, our DENV-1 validation provides proof-of-concept evidence warranting further investigation.

Our proteomic analysis extends dengue studies by Kumar et al. (37) and Han et al. (38), revealing thromboinflammatory signatures amplified by cross-reactive antibodies. While previous studies have identified DHF biomarkers, our dual-seropositive patients showed overlapping proteins and unique TGF-β signaling and complement activation, highlighting novel ADE–autoantibody synergy, where SARS-CoV-2 exposure creates vascular leakage via THBS1 and STXBP5 as “second hits”. This demonstrates heterologous viral imprinting, creating a “hybrid” dengue phenotype combining DHF’s acute vascular pathology with long COVID’s chronic inflammation through Th2 skewing and IL-6-linked ADE (41).

It is important to note that, as a hypothesis-generating study, our proteomic analysis (279 proteins and 18 DEPs) and ADE assays provide novel insights into SARS-CoV-2–dengue interactions, revealing group-level dysregulation in the context of acute dengue infection stratified by serostatus, rather than individual-level risk prediction. Despite the small sample size (n = 18) limiting generalizability, this approach enabled deep profiling in a resource-limited setting and revealed candidate biomarkers (e.g., thrombospondin-1 and STXBP5) and pathways (e.g., complement and TGF-β) for severe dengue. The absence of extensive comorbidity screening and the pooled proteomic design mean that these findings should be interpreted as hypothesis-generating and require validation in larger, individually phenotyped cohorts with comprehensive clinical and metabolic characterization. Unlike studies focusing solely on primary dengue, our findings suggest a hybrid phenotype in dual-seropositive patients, blending DHF-like vascular pathology with immune dysregulation similar to that observed in long COVID. The unique phagosome pathway in NS1/SARS-DENV_IgG suggests enhanced opsonization, justifying the need for further mechanistic studies. The pathway-level enrichment in complement/coagulation cascades and TGF-β signaling observed in the dual-seropositive group is consistent with ADE mechanisms and warrants further investigation; however, confounding by metabolic or other unmeasured clinical factors cannot be definitively excluded at this stage. Additional limitations include the lack of longitudinal data and extended cytokine profiling (e.g., IL-4/IL-10), although markers such as IL-6/CRP and ADE assays with other serotypes support Th2 polarization. Future research should aim to validate these biomarkers in larger cohorts and explore therapeutics targeting platelet activation or Th2 cytokines, as recommended by the WHO’s call for integrated arbovirus surveillance. Furthermore, our results highlight a new concern regarding the effects of SARS-CoV-2 vaccination, particularly in dengue-endemic regions of the world.

## Conclusion

This pilot study shows that prior SARS-CoV-2 exposure modulates dengue pathogenesis in a Mexican cohort, as proteomic profiling of 279 high-confidence proteins revealed enriched complement/coagulation cascades and TGF-β signaling in dual-seropositive patients. Differentially expressed proteins, including thrombospondin-1 and STXBP5, suggest Th2-polarized immunity and ADE-like mechanisms, supported by enhanced dengue virus replication *in vitro* with anti-SARS-CoV-2 sera. These findings highlight the role of cross-reactive antibodies in exacerbating dengue severity, offering biomarkers for risk stratification in the Americas, where dengue cases have surged post-COVID-19 (PAHO, 2023–2025).

## Limitations of the study

This pilot study was designed as a hypothesis-generating investigation with a small sample size (n = 18), which is appropriate for exploratory proteomic profiling in a resource-limited setting. The sample size reflects practical constraints of the endemic region and was powered to identify broad pathway-level dysregulation rather than individual-level biomarkers. Statistical power was not formally calculated for this exploratory pilot study; future validation studies will require formal power analysis based on effect sizes derived from these preliminary findings.

## Data Availability

The original contributions presented in the study are included in the article/**Supplementary Material**. Further inquiries can be directed to the corresponding authors.

## Supplementary material

Supplementary Figure 1 SDS-PAGE verification of serum protein integrity across study cohorts. Representative 12% SDS-PAGE gel stained with Coomassie Blue showing serum protein profiles from individual participants. Lanes are labeled with corresponding sample IDs. Control Group (Ctrl 1-3), Group 1 (NS1/SARS_IgG), Group 2 (NS1/SARS-DENV_IgG), and Group 3 (NS1/DENV_IgG) demonstrate consistent protein banding patterns, including prominent albumin (~66 kDa) and IgG heavy/light chains (~50 kDa and ~25 kDa). The uniform band intensity and absence of low molecular weight smears confirm high sample quality and lack of degradation prior to proteomic processing. Molecular weight markers (kD) are indicated on the left.

Supplementary Figure 2 KEGG Pathway Enrichment Analysis of Differentially Expressed Proteins in the NS1/SARS-DENV_IgG Cohort. Bubble plot showing the top dysregulated KEGG pathways identified in dual-seropositive dengue patients (NS1/SARS-DENV_IgG group). The x-axis represents fold enrichment of pathway members, while bubble size indicates the number of genes involved (N. of Genes = 1; shown in black). Pathway significance is marked by -log10(FDD) values. Complement and coagulation cascades (fold enrichment ~55) and TGF-β signaling pathway (fold enrichment ~50) show the strongest dysregulation, both mechanistically linked to severe dengue pathogenesis. Additional enriched pathways include ECM-receptor interaction, P53 signaling, malaria, and asthma, suggesting multi-system immune dysregulation.
