# Supplementary figures and images for: Pilot proteomic analysis of immune dysregulation in dengue with prior SARS-CoV-2 infection
Source: Front Immunol. 2026 Jan 8;16:1696179. doi: 10.3389/fimmu.2025.1696179 (PMC12823978; doi:10.3389/fimmu.2025.1696179)

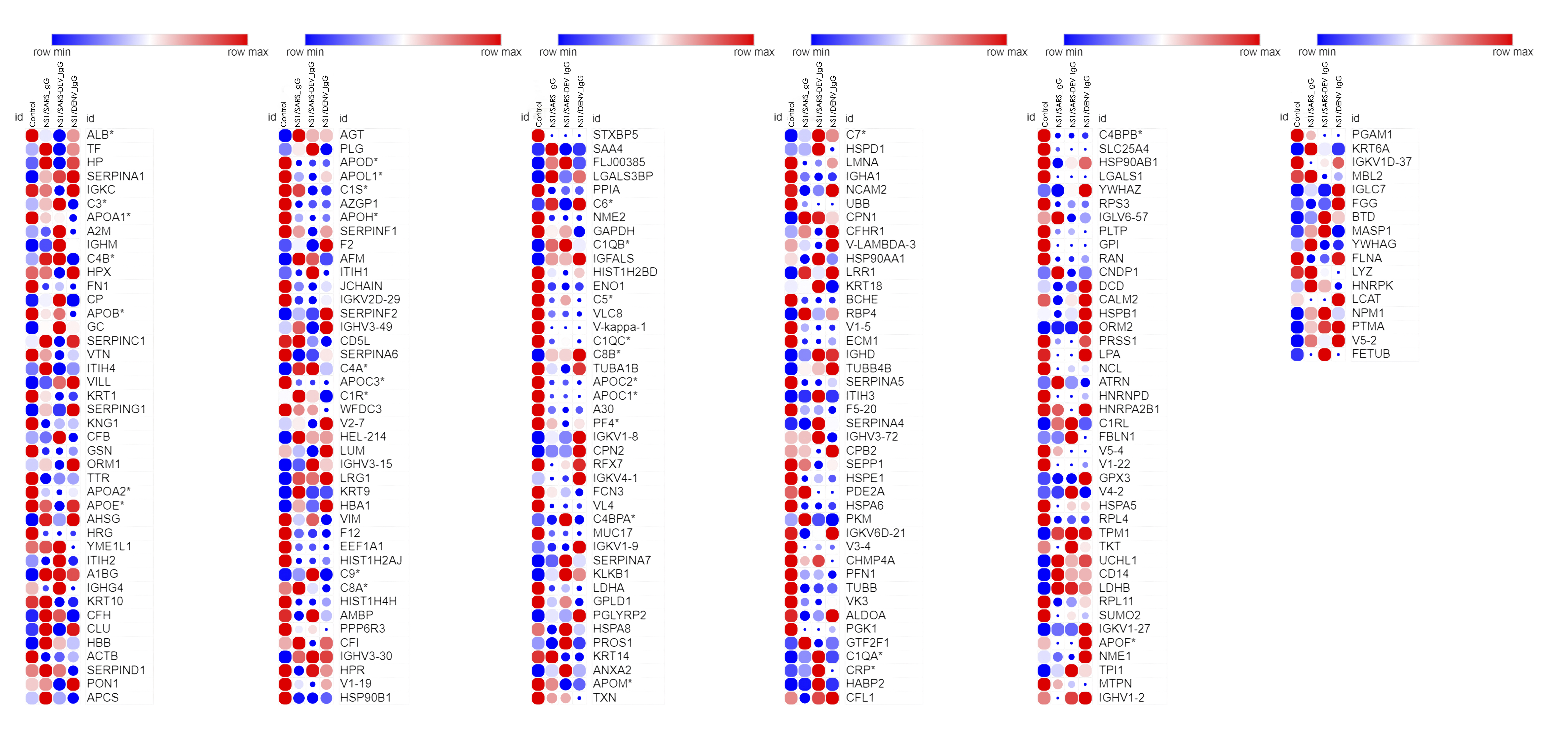

Supplement: Supplementary Figure 1 — SDS-PAGE verification of serum protein integrity across study cohorts. Representative 12% SDS-PAGE gel stained with Coomassie Blue showing serum protein profiles from individual participants. Lanes are labeled with corresponding sample IDs. Control Group (Ctrl 1-3), Group 1 (NS1/SARS_IgG), Group 2 (NS1/SARS-DENV_IgG), and Group 3 (NS1/DENV_IgG) demonstrate consistent protein banding patterns, including prominent albumin (~66 kDa) and IgG heavy/light chains (~50 kDa and ~25 kDa). The uniform band intensity and absence of low molecular weight smears confirm high sample quality and lack of degradation prior to proteomic processing. Molecular weight markers (kD) are indicated on the left. [file Image1.jpg]

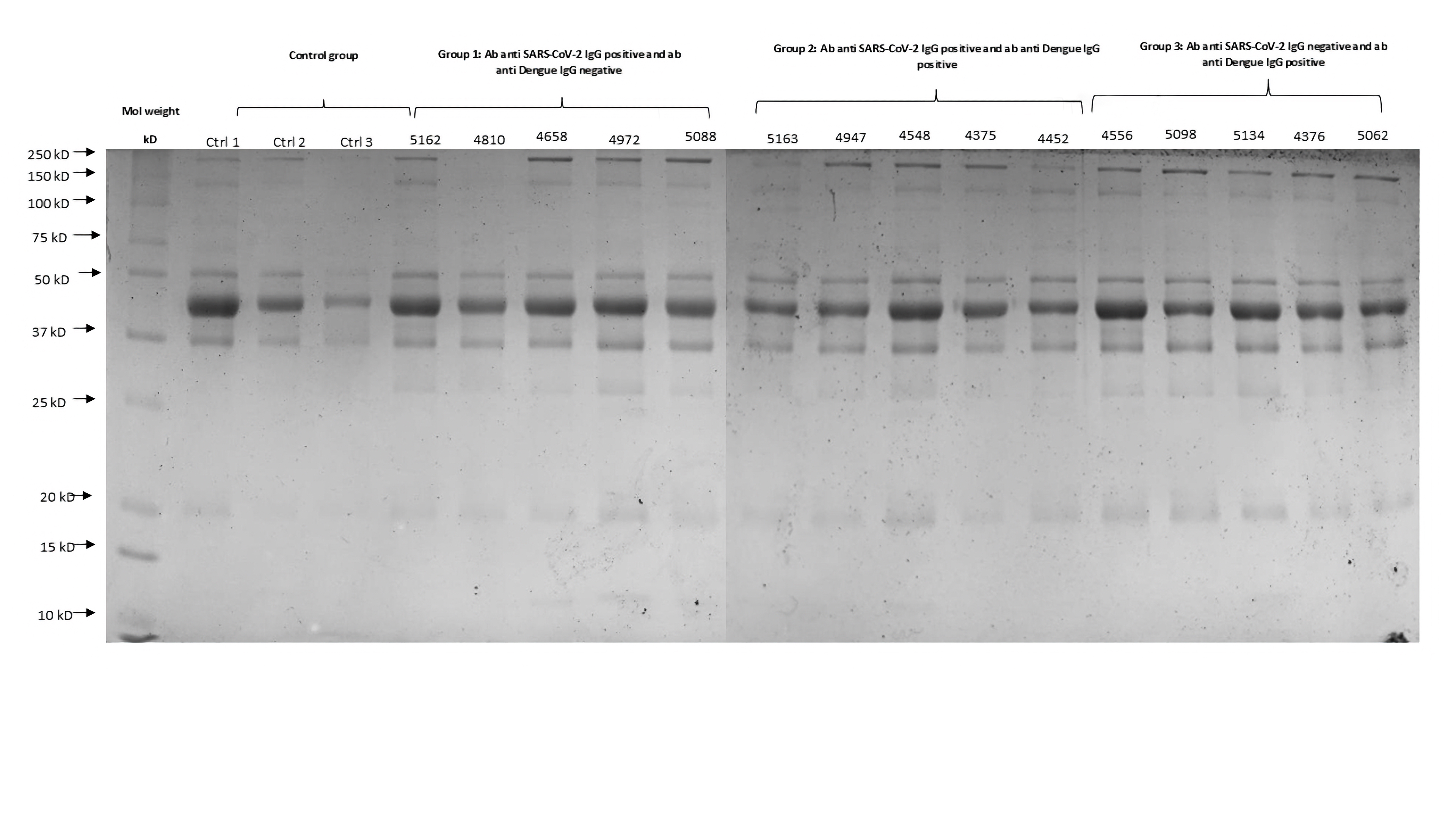

Supplement: Supplementary Figure 2 — KEGG Pathway Enrichment Analysis of Differentially Expressed Proteins in the NS1/SARS-DENV_IgG Cohort. Bubble plot showing the top dysregulated KEGG pathways identified in dual-seropositive dengue patients (NS1/SARS-DENV_IgG group). The x-axis represents fold enrichment of pathway members, while bubble size indicates the number of genes involved (N. of Genes = 1; shown in black). Pathway significance is marked by -log10(FDD) values. Complement and coagulation cascades (fold enrichment ~55) and TGF-β signaling pathway (fold enrichment ~50) show the strongest dysregulation, both mechanistically linked to severe dengue pathogenesis. Additional enriched pathways include ECM-receptor interaction, P53 signaling, malaria, and asthma, suggesting multi-system immune dysregulation. [file Image2.jpeg]
